# Analysis of pediatric surgical trauma cases in a tertiary care center

**DOI:** 10.1186/1865-1380-7-S1-P10

**Published:** 2014-07-25

**Authors:** Anjan Kumar Dhua, Lavanya Chellam, Manoj Joshi

**Affiliations:** 1 Pondicherry Institute of Medical Sciences, Pondicherry, India

## Objective

To evaluate the pattern of pediatric trauma cases and their subsequent morbidity and mortality.

## Methods

Retrospective review of all cases (less than 12 years) referred to the pediatric surgery unit of a teaching hospital over a period of 26 months (October 2011 to November 2013). Burns, isolated head injuries, ophthalmic injuries, and orthopedic injuries were excluded. The following parameters were assessed: age group, sex, mode of trauma, type of injury, place where the trauma occurred, time interval between injury and presentation and the overall morbidity as well as mortality.

## Results

A total of 194 cases of trauma were assessed excluding 24 cases in whom the details were incomplete. Maximum number of cases (n=84) involved the children in school going age. The male to female ratio was 2.3:1. The majority of the cases (74%) were trivial trauma. Both road (36.5%) and home (35%) were almost equally unsafe in our series. The rest (28.3%) of the injuries occurred in the school/playground. The median time interval in presentation was 3 hours (ranges from 30 min to 7 days). Fourteen patients (7.2%) required operative intervention and among them none had any major complications until last follow up. There was no mortality in the series.

## Limitations

The study is based on hospital data and hence the injury patterns and results cannot be generalized for the entire community.

## Conclusion

The majority of the pediatric traumas are preventable. More coordinated efforts are needed to make our roads and home a safer place. Response time and referral pattern needs to be addressed to decrease the delay in reaching.

